# Two cases of a non-progressive hepatic form of glycogen storage disease type IV with atypical liver pathology

**DOI:** 10.1016/j.ymgmr.2020.100601

**Published:** 2020-05-18

**Authors:** Keiko Ichimoto, Tomoo Fujisawa, Masaru Shimura, Takuya Fushimi, Makiko Tajika, Ayako Matsunaga, Minako Ogawa-Tominaga, Nana Akiyama, Yuki Naruke, Hiroshi Horie, Tokiko Fukuda, Hideo Sugie, Ayano Inui, Kei Murayama

**Affiliations:** a Center for Medical Genetics, Department of Metabolism, Chiba Children's Hospital, 579-1 Heta-cho, Midori-ku, Chiba 266-0007, Japan; b Department of Pediatric Hepatology and Gastroenterology, Saiseikai Yokohama-shi Tobu Hospital, 3-6-1 Shimosueyoshi, Tsurumi-ku, Yokohama 230-8765, Japan; c Department of Pathology, Chiba Children's Hospital, 579-1 Heta-cho, Midori-ku, Chiba 266-0007, Japan; d Department of Pediatrics, Hamamatsu University School of Medicine, 1-20-1 Handayama, Higashi-ku, Hamamatsu 431-3192, Japan; e Faculty of Health and Medical Sciences, Tokoha University, 1230 Miyakodachou, Kita-ku, Hamamatsu 431-2102, Japan

**Keywords:** Andersen disease, *GBE1*, GSD IV, M2BPGi, Nutrition therapy, GSD IV, Glycogen storage disease type IV, PAS, periodic acid-Schiff, GBE, glycogen-branching enzyme, SD, standard deviation, AST, aspartate transaminase, ALT, alanine aminotransferase, γ-GTP, gamma-glutamyltransferase, M2BPGi, Mac-2 binding protein glycosylation isomer, COI, cut-off index, PAS-D, periodic acid-Schiff-diastase

## Abstract

Glycogen storage disease type IV (GSD IV) is a rare inborn metabolic disorder characterized by the accumulation of amylopectin-like glycogen in the liver or other organs. The hepatic subtype may appear normal at birth but rapidly develops to liver cirrhosis in infancy. Liver pathological findings help diagnose the hepatic form of the disease, supported by analyses of enzyme activity and *GBE1* gene variants. Pathology usually shows periodic acid-Schiff (PAS) positive hepatocytes resistant to diastase. We report two cases of hepatic GSD IV with pathology showing PAS positive hepatocytes that were mostly digested by diastase, which differ from past cases. Gene analysis was critical for the diagnosis. Both cases were found to have the same variants c.288delA (p.Gly97GlufsTer46) and c.1825G > A (p.Glu609Lys). These findings suggest that c.1825G > A variant might be a common variant in the non-progressive hepatic form of GSD IV.

## Highlights

• GSD IV is caused by homozygous/compound heterozygous mutations in *GBE1.*
• Pathological features of GSDIV are PAS positive hepatocytes digested by PAS-D.
• Some non-progressive hepatic GSD IV cases had hepatocytes mostly digested by PAS-D.
• Gene analysis was used for diagnosis and prognosis prediction.
• Both cases had c.1825G > A *GBE1*, indicating a role in non-progressive hepatic GSD IV.

## 1 Introduction

Glycogen storage disease type IV (GSD IV; Andersen disease; OMIM #232500) is a clinically heterogeneous disorder caused by homozygous or compound heterozygous variants in the *GBE1* gene, located on chromosome 3p12, which encodes glycogen-branching enzyme (GBE). The classic hepatic form of GSD IV, first reported by Andersen in 1956 [1], starts in early childhood and progresses to lethal cirrhosis by five years of age, while the non-progressive hepatic form does not progress to liver cirrhosis [2]. The neuromuscular form of GSD IV is classified by age at onset [3]. GBE is responsible for adding short glucosyl chains to the growing glycogen molecule to form its branched polymeric structure. GBE deficiency leads to the accumulation of abnormal glycogen molecules with fewer branch points and longer outer branches with an amylopectin-like structure. This abnormal glycogen accumulates in the liver, muscle, heart, and central and peripheral nervous systems [4]. Light microscopy revealed liver pathology with enlarged hepatocytes containing centrally placed glycogen deposits that stained strongly positive with PAS and typically showed resistance to diastase [2].

Because GSD IV is rare glycogen storage disease, it is difficult to investigate its liver pathology. We report two cases of non-progressive hepatic GSD IV with liver pathology showing PAS positive deposits that were mostly digested by diastase. Gene analysis was important for the diagnosis and prognosis prediction. We also reviewed the long-term clinical courses of previously reported patients with non-progressive hepatic GSD IV.

## 2 Case report

### 2.1 Case 1

**Fig. 1:**
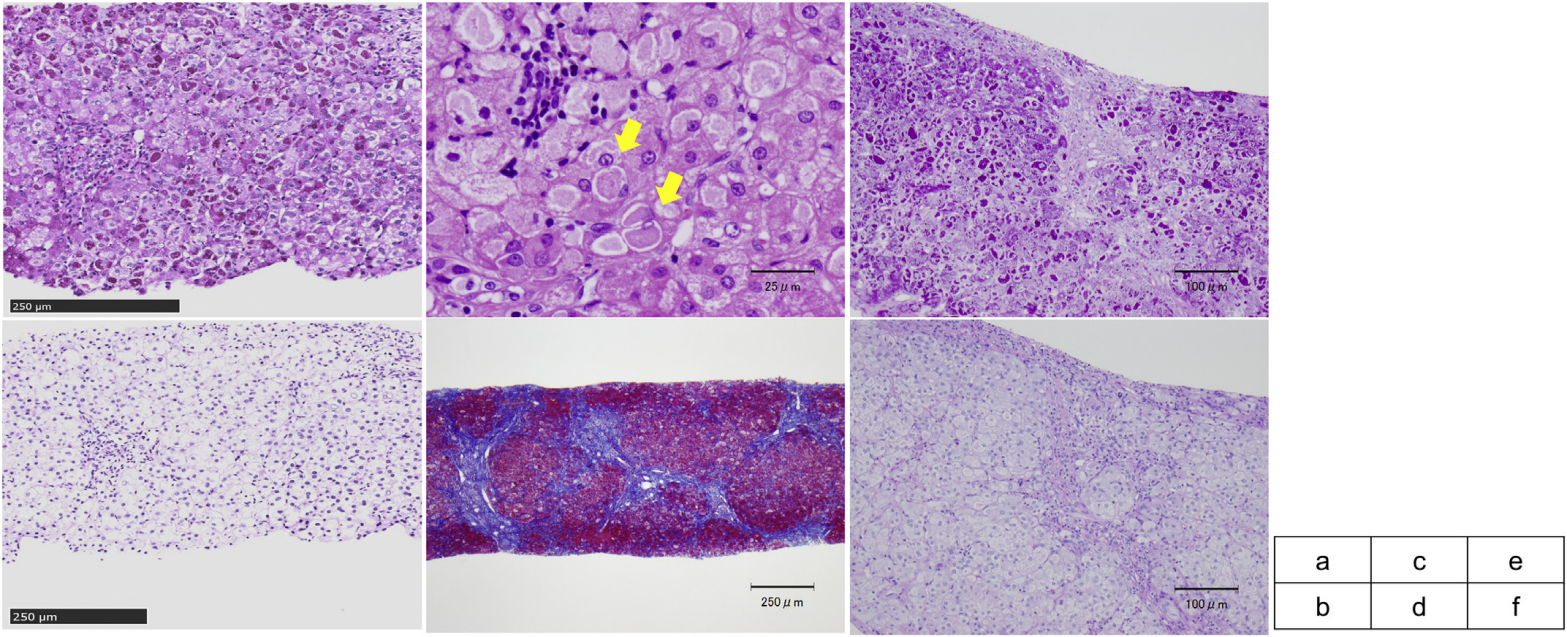
Light microscopic liver pathology. a: Case 1, periodic acid-Schiff (PAS) staining. b: Case 1, periodic acid-Schiff-diastase (PAS-D) staining. Hepatocytes were stained with PAS and were mostly digested by PAS-D. c: Case 2, hematoxylin and eosin staining. Eosinophilic materials surrounded by a halo (indicated by arrows). d: Case 2, Masson staining. Bridging fibrosis was observed. e: Case 2, PAS staining. f: Case 2, PAS-D staining. Hepatocytes were stained with PAS and were mostly digested by PAS-D.

**Fig. 2:**
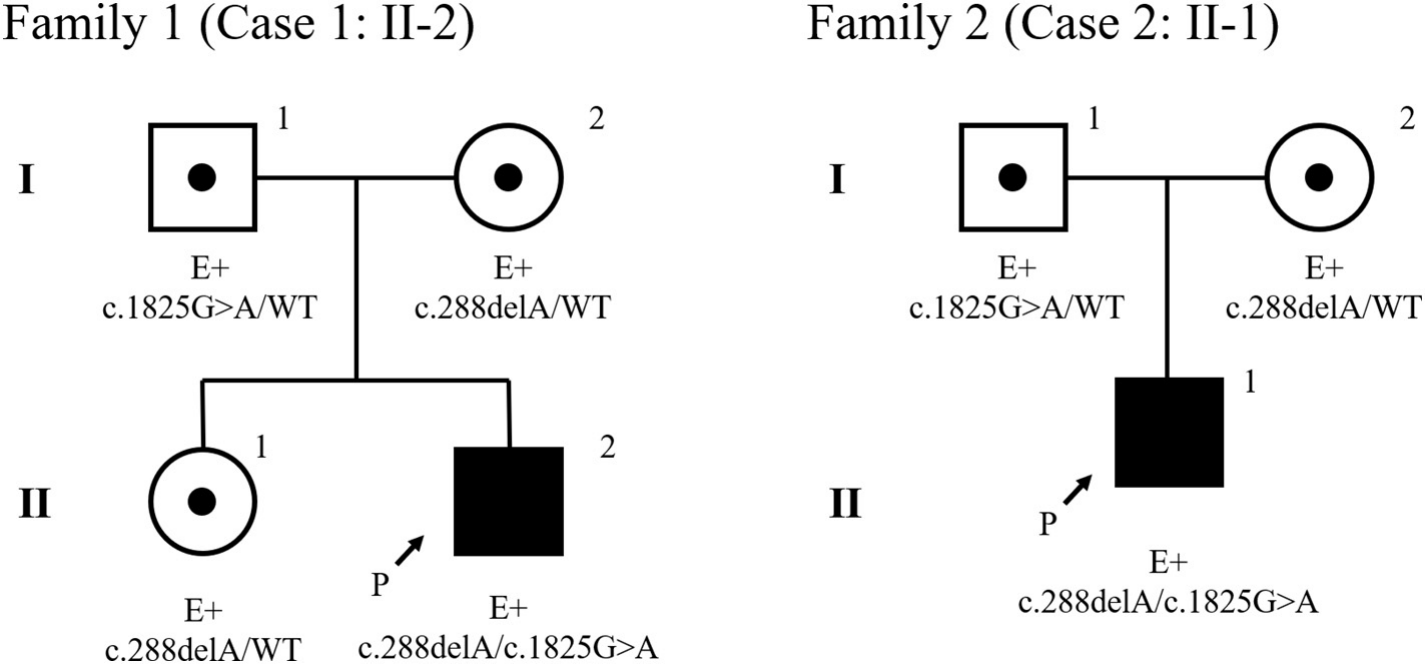
Pedigrees of the two families. Both cases had a compound heterozygous variant. The probands are indicated by arrows. Sanger sequencing revealed each parent, and elder sister in case 1, had a heterozygous *GBE1* varinat [NM_000158.4].

A Japanese male infant was born at 39 weeks gestation, with a birth weight of 3277 g. He was the second child of non-consanguineous parents. He had mild speech delay and was receiving speech therapy. Liver dysfunction was detected at 3 years of age and he was referred to a pediatric hepatologist. He weighed 17.2 kg (+1.8 standard deviation (SD)) and was 96.2 cm (+0.1 SD) tall. He showed no motor development delay. His liver and spleen were not palpable below the costal margin, and there were no neuromuscular symptoms or signs. Laboratory studies showed elevated transaminases: aspartate transaminase (AST), 204 IU/L; alanine aminotransferase (ALT), 153 IU/L; and gamma-glutamyltransferase (γ-GTP) 84 IU/L). Total bile acid was normal (7.9 μmol/L). Abdominal ultrasound showed a slightly bright liver. Liver biopsy was performed at 3 years and 5 months (Fig. 1a, b), and pathological analysis showed enlarged hepatocytes with an accumulation of PAS positive eosinophilic materials surrounded by a halo. Whole-exome sequencing was performed and *GBE1* variants were revealed. Direct sequencing of the *GBE1* gene revealed c.288delA (p.Gly97GlufsTer46) and c.1825G > A (p.Glu609Lys) missense variant. The c.288delA variant was also present in the patient's sister and mother, and the c.1825G > A mutation was also present in his father (Fig. 2, Family 1). Erythrocyte GBE activity was 0.2 μmol Pi/min/g Hb, approximately 5% of that in two healthy adult controls (4.0 ± 0.2 μmol Pi/min/g Hb).

The patient was healthy at 5 years of age, with AST 42 IU/L and ALT 30 IU/L. Serum Mac-2 binding protein glycosylation isomer (M2BPGi), a novel serum diagnostic marker for liver fibrosis, had a cut-off index (COI) of 1.13 at 3 years and remained high at 1.34 at 5 years.

### 2.2 Case 2

A Japanese male infant was born at 38 weeks and 4 days gestation, with a birth weight of 3100 g. He was the first child of non-consanguineous parents. His growth and development were normal, but abdominal distension was noted at 1 year 8 months of age. Elevated liver transaminase levels were observed (AST, 644 IU/L; ALT, 360 IU/L), and he was referred to our hospital at 2 years of age. He weighed 12.1 kg (+0.4 SD) and was 84.1 cm (−0.4 SD) tall. The liver was palpable eight fingers below the right costal margin and the edge was slightly hard. The spleen was palpable four fingers below the left costal margin. There were no neuromuscular symptoms or signs. Laboratory analyses showed elevated transaminases (AST, 479 IU/L; ALT, 217 IU/L, and γ-GTP, 163 IU/L), total bile acid (47.3 μmol/L), and a slightly decreased platelet count (152,000/μL). The prothrombin time was normal (12 s). Abdominal ultrasound revealed hepatosplenomegaly and high intensity in the liver. Liver biopsy was performed at 2 years 3 months of age. Pathological analysis showed half of the hepatocytes were enlarged with an accumulation of eosinophilic materials surrounded by a halo, and moderate inflammatory infiltration in the portal area (Fig. 1c–f). Because of the bridging fibrosis, the classic hepatic form of GSD IV was suspected. The erythrocyte GBE activity was 0.2 μmol Pi/min/g Hb, approximately 7% of the mean activity in two healthy adult controls (2.85 ± 0.45 μmol Pi/min/g Hb). Next-generation sequencing of the *GBE1* gene revealed two heterozygous variants c.288delA (p.Gly97GlufsTer46) and c.1825G > A (p.Glu609Lys) which was also present in his mother and father, respectively (Fig. 2, Family 2).

Hypoglycemia was not observed after fasting or on sick days. At 2 years 4 months of age, the serum zinc level was 49 μg/dL (normal >80 μg/dL); therefore, oral zinc was prescribed. He started a high-protein diet with carbohydrate restriction, and his liver stiffness softened after a few months. Furthermore, his serum M2BPGi level decreased rapidly from 2.14 and 2.57 COI at 2 years 3 months and 2 years 5 months, respectively, to a normal level of 0.99 COI at 2 years 10 months. His hepatic condition remained stable at 1 year after diagnosis and his growth was normal.

## 3 Discussion

Here, we report two cases of non-progressive hepatic GSD IV, in which liver pathology showed PAS-stained hepatocytes that were mostly digested by periodic acid-Schiff-diastase (PAS-D). This finding is not usual in GSD IV, and partial digestion previously reported in the non-progressive hepatic form of disease (Table 1, Cases 3–7) might be associated with residual enzyme activity. The PAS positive material digested by PAS-D resembled Lafora bodies. Furthermore, Case 1 had a mild speech delay, which could be a symptom used for the differential diagnosis of Lafora disease, a neurological disorder with onset in school aged children to teenagers. It is characterized by progressively worsening seizures, dementia, and myoclonic attacks, and morphologically by the presence of large PAS-positive intraneural inclusions known as Lafora bodies, which are typically found in the brain, liver, skeletal and cardiac myocytes, eccrine duct, and apocrine myoepithelial cells of sweat glands [5]. However, GSD IV and Lafora disease can be distinguished from each other by their clinical features, with confirmation by enzyme activity and genetic analysis.

**Table 1:** Clinical features and liver pathology of the non-progressive hepatic form of GSD IV. Cases 1–3 are Japanese.

Case	Sex	Age of onset	Symptoms							Liver biopsy	
			Failure to thrive	Increased transaminases	Hepatomegaly	Splenomegaly	Muscle	Heart	Neuron	Age of biopsy	Pathology
1	M	3 y	−	+	−	−	−	−	−	41 m	PAS-positive deposits in hepatocytes mostly digested by PAS-D; mild fibrotic changes
2	M	20 m	−	+	+	+	−	−	−	27 m	PAS-positive deposits in hepatocytes mostly digested by PAS-D moderate fibrotic changes with bridging fibrosis
3 [9]	M	2 y	−	+	+	+	−	−	−	2 y	PAS-positive deposits in hepatocytes partially resistant to PAS-D; mild fibrotic changes
4 [11,12]	F	12 m	+	+	+	−	−	−	−	18 m	PAS-positive deposits in hepatocytes partially resistant to PAS-D; portal areas broadened with advanced fibrosis
5 [11,12]	M	9 m	−	+	+	+(2.5y)	−	−	−	2.5 y	PAS-positive deposits in hepatocytes partially resistant to PAS-D
6 [2,12]	M	23 m	−	+	+	+	−	−	−	27 m 38 m	PAS-positive deposits in hepatocytes resistant to PAS-D; advanced fibrosis seen at 38 m
7 [12]	M	36 m	+	+	+	−	−	−	−	4 y	PAS-positive deposits in hepatocytes partially resistant to PAS-D
8 [[16]]	M	9 m	−	+	+	−	−	−	−	26 m	PAS-positive deposits in hepatocytes; not referred for PAS-D; moderate fibrotic changes

M: male, F: female, m: months, y: years.

Surprisingly, both current unrelated cases had the same variants. The *GBE1* gene was first isolated in 1993 [6]. The non-progressive hepatic form of GSD IV is rare and few genetic studies have been reported. The *GBE1* variants c.288delA and c.1825G > A were detected in the current cases. The c.288delA homozygous variant was reported previously in a newborn female infant with severe hypotonia and dilated cardiomyopathy [7]. Hypoglycemia worsened at 4 months of age and hepatomegaly was observed. Her ALT levels were < 100 IU/L and total bilirubin levels were normal. She died of cardiomyopathy at 4 months of age. Liver pathology was not performed. Two cases of the congenital neuromuscular form of GSD IV with the c.288delA variant in one allele were also reported [8].

The c.1825G > A variant was recently reported in a Japanese patient with non-progressive hepatic GSD IV, who had compound heterozygous *GBE1* variants c.137A > C (p.Gln46Pro)/c.1825G > A (p.Glu609Lys) [9]. His clinical features are presented in Table 1, Table 2 (Case 3). In this report [9], the functional analysis of mutant GBE proteins was performed using *Escherichia coli* BL21 (DE3) cells, and the branching activity of each protein was measured using the amylose‑iodine absorbance spectrum as described previously [10]. The enzyme activities of c.137A > C and c.1825G > A were 1.21 U/mg and 1.15 U/mg, respectively, which were both lower than that of wild-type GBE (2.18 U/mg). The activity of the c.1825G > A variant was almost half that of the normal activity, and this level was reported to be important for the clinical features of non-progressive hepatic GSD IV. The variant c.986A > C allele, resulting in half-normal activity, was also associated with non-progressive hepatic GSD IV [11]. Although GBE activity remains low, most cases of the non-progressive hepatic form of disease develop normal liver transaminase levels or liver size in later years [12]. However, the mechanism responsible for this reversible course is unclear. Both of our cases and one previous Japanese case [9] with the same c.1825G > A *GBE1* gene variant were diagnosed as the non-progressive hepatic form. Initially, it is difficult to distinguish between the non-progressive hepatic form or classic hepatic form of GSD IV using GBE activity alone. Under these circumstances, the most effective treatment for the classic hepatic form is liver transplantation [13]. The long-term course of non-progressive hepatic GSD IV was previously reported (Table 1, Table 2) [2,9,11,12] and showed onset ages of around 1 year to 3 years, and hepatopathy had recovered by school age in most cases. Therefore, the c.1825G > A variant has a potentially good prognosis and cases with this variant might avoid unnecessary liver transplantation.

**Table 2:** GBE activity, *GBE1* gene variant [NM_000158.4] and clinical findings of the non-progressive hepatic form of GSD IV. Case 8 had 0% liver GBE activity.

Case	GBE activity (%control)	*GBE1* gene mutation	Complications; long-term clinical findings	Age at reporting
1	9% (erythro)	c.288delA (p.Gly97GlufsTer46)/c.1825G ≥A (p.Glu609Lys)	Mild speech delay	5 y
2	7% (erythro)	c.288delA (p.Gly97GlufsTer46)/c.1825G ≥A (p.Glu609Lys)	None	3 y
3 [9]	11% (erythro)	c.137A > C (p.Gln46Pro)/c.1825G ≥A (p.Glu609Lys)	At 5 y transaminase normalized; at 8 y hepatosplenomegaly disappeared; at 13 y epilepsy	17 y
4 [11,12]	8% (fibro)	c.671 T > C(p.Leu224Pro)/c.986A > C (p.Tyr329Ser)	At 29 m transaminase normalized and no hepatosplenomegaly	3 y 8 m
5 [11,12]	2.5 y: 10–12% (fibro); 20 y: 13% (fibro)	c.986A > C (p.Tyr329Ser)/not referred	At 10 y transaminase normalized; at 16 y no hepatosplenomegaly; at 20 y fibroblast GBE activity was low	20 y
6 [2,12]	8% (liver) 13% (fibro)	Not referred	At 38 m fasting hypoglycemia; at 4 y liver biopsy performed, fibrosis not advanced; liver GBE activity was half of normal; at 60 m transaminase normalized; at 13 y splenomegaly remained	13 y
7 [12]	9% (fibro)	Not referred	None	5 y
8 [16]	0% (liver) 13% (fibro) 5–20% (leuko)	c.691+2T > C/c.1583A > G	After diagnosis mild microalbuminuria revealed, treated by ACE inhibitor; renal complication did not progress; at 17 y no hepatomegaly and liver function normal; normal growth	17 y

m: months, y: years, erythro: erythrocytes, fibro: fibroblasts, leuko: leukocytes.

Nutritional management strategies for GSD IV have not been established, but a few cases have been reported [12,[14], [15], [16]]. A patient with moderate fibrosis at 26 months of age was treated with a high-protein diet with a restriction of non-utilizable sugars and regular food intake. His liver function became normal and he was healthy at 17 years of age [16]. Conversely, four patients with non-progressive hepatic GSD IV were all maintained with normal nutrition and subsequently recovered normal liver function [12]. In Case 2, a high-protein diet with carbohydrate restriction was initiated and liver stiffness improved after a few months. Furthermore, M2BPGi decreased rapidly. Whether M2BPGi is a good marker for liver fibrosis in glycogen storage diseases is unknown. Case 2 suggests that a carbohydrate restricted diet may be effective in some cases with no side effects. Further studies are expected to explore this issue.

## 4 Conclusion

We report two cases of non-progressive hepatic GSD IV with atypical liver pathology. They showed PAS-stained hepatocytes that were mostly digested by PAS-D. Genetic analyses played an important role in the diagnosis. We suggest that the c.1825G > A *GBE1* variant may be a causative factor in the non-progressive hepatic form of GSD IV and might avoid unnecessary liver transplantation in the early stage of disease.

## Ethical consideration

This study was performed in accordance with the Helsinki Declaration. The patients and parents in our study provided permission to publish the features of their cases, and the identities of the patients have been protected.

## Funding source

This research did not receive any specific grants from funding agencies in the public, commercial, or not-for-profit sectors.

## Declaration of Competing Interest

All the authors declare that they have no conflicts of interest to disclose.

## References

[1] Andersen D.H. (1956). Familial cirrhosis of the liver with storage of abnormal glycogen. Lab. Investig..

[2] Greene H.L., Brown B.I., McClenathan D.T., Agostini R.M., Taylor S.R. (1988). A new variant of type IV glycogenosis: deficiency of branching enzyme activity without apparent progressive liver disease. Hepatology.

[3] Bruno C., van Diggelen O.P., Cassandrini D., Gimpelev M., Giuffrè B., Donati M.A., Introvini P., Alegria A., Assereto S., Morandi L., Mora M., Tonoli E., Mascelli S., Traverso M., Pasquini E., Bado M., Vilarinho L., van Noort G., Mosca F., DiMauro S., Zara F., Minetti C. (2004). Clinical and genetic heterogeneity of branching enzyme deficiency (glycogenosis type IV). Neurology..

[4] Lamperti C., Salani S., Lucchiari S., Bordoni A., Ripolone M., Faqiolari G., Fruquqlietti M.E., Cruqnola V., Colombo C., Cappellini A., Prelle A., Bresolin N., Comi G.P., Moqqio M. (2009). Neuropathological study of skeletal muscle, heart, liver, and brain in a neonatal form of glycogen storage disease type IV associated with a new mutation in GBE1 gene. J. Inherit. Metab. Dis..

[5] Turnbull J., Tiberia E., Striano P., Genton P., Carpenter S., Ackerley C.A., Minassian B.A. (2016). Lafora disease. Epileptic Disord..

[6] Thon V.J., Khalil M., Cannon J.F. (1993). Isolation of human glycogen branching enzyme cDNAs by screening complementation in yeast. J. Biol. Chem..

[7] Nambu M., Kawabe K., Fukuda T., Okuno T.B., Ohta S., Nonaka I., Sugie H., Nishino I. (2003). A neonatal form of glycogen storage disease type IV. Neurology..

[8] Li S.C., Hwu W.L., Lin J.L., Bali D.S., Yang C., Chu S.M., Chien Y.H., Chou H.C., Chen C.Y., Hsieh W.S., Tsao P.N., Chen Y.T., Lee N.C. (2012). Association of the congenital neuromuscular form of glycogen storage disease type IV with a large deletion and recurrent frameshift mutation. J. Child Neurol..

[9] Iijima H., Iwano R., Tanaka Y., Muroya K., Fukuda T., Sugie H., Kurosawa K., Adachi M. (2018). Analysis of GBE1 mutations via protein expression studies in glycogen storage disease type IV: a report on a non-progressive form with a literature review. Mol. Genet. Metab. Rep..

[10] Krisman C.R., Tolmasky D.S., Raffo S. (1985). Branching enzyme assay: selective quantitation of the alpha 1,6-linked glucosyl residues involved in the branching points. Anal. Biochem..

[11] Bao Y., Kishnani P., Wu J.Y., Chen Y.T. (1996). Hepatic and neuromuscular forms of glycogen storage disease type IV caused by mutations in the same glycogen-branching enzyme gene. J. Clin. Invest..

[12] McConkie-Rosell A., Wilson C., Piccoli D.A., Boyle J., DeClue T., Kishnani P., Shen J.J., Boney A., Brown B., Chen Y.T. (1996). Clinical and laboratory findings in four patients with the non-progressive hepatic form of type IV glycogen storage disease. J. Inherit. Metab. Dis..

[13] Davis M.K., Weinstein D.A. (2008). Liver transplantation in children with glycogen storage disease: controversies and evaluation of the risk/benefit of this procedure. Pediatr. Transplant..

[14] Greene H.L., Ghishan F.K., Brown B., McClenathan D.T., Freese D. (1988). Hypoglycemia in type IV glycogenosis: hepatic improvement in two patients with nutritional management. J. Pediatr..

[15] Levin B., Burgess E.A., Mortimer P.E. (1968). Glycogen storage disease type IV, amylopectinosis. Arch. Dis. Child..

[16] Szymańska E., Szymańska S., Truszkowska G., Ciara E., Pronicki M., Shin Y.S., Podskarbi T., Kępka A., Śpiewak M., Płoski R., Bilińska Z.T., Rokicki D. (2018). Variable clinical presentation of glycogen storage disease type IV: from severe hepatosplenomegaly to cardiac insufficiency. Some discrepancies in genetic and biochemical abnormalities. Arch. Med. Sci..

